# An Inflammatory Landscape for Preoperative Neurologic Deficits in Glioblastoma

**DOI:** 10.3389/fgene.2019.00488

**Published:** 2019-06-04

**Authors:** Amal Katrib, Hyun-Hwan Jeong, Nina L. Fransen, Kristin S. Henzel, Jeremy A. Miller

**Affiliations:** ^1^Department of Microbiology, Immunology and Molecular Genetics, University of California, Los Angeles, Los Angeles, CA, United States; ^2^Institute for Systems Biology, Seattle, WA, United States; ^3^Department of Molecular and Human Genetics, Baylor College of Medicine, Houston, TX, United States; ^4^Jan and Dan Duncan Neurological Research Institute, Texas Children’s Hospital, Houston, TX, United States; ^5^Department of Neuroimmunology, Netherlands Brain Bank, Netherlands Institute for Neuroscience, Amsterdam, Netherlands; ^6^German Center for Neurodegenerative Diseases, Bonn, Germany; ^7^Allen Institute for Brain Science, Seattle, WA, United States

**Keywords:** glioblastoma, cancer, neurologic deficit, inflammation, mesenchymal subtype, RNA sequencing, interleukin 1

## Abstract

**Introduction:** Patients with glioblastoma (GBM), one of the most aggressive forms of primary brain tumors, exhibit a wide range of neurologic signs, ranging from headaches to neurologic deficits and cognitive impairment, at first clinical presentation. While such variability is attributed to inter-individual differences in increased intracranial pressure, tumor infiltration, and vascular compromise, a direct association with disease stage, tumor size and location, edema, and necrotic cell death has yet to be established. The lack of specificity of neurologic symptoms often confounds the diagnosis of GBM. It also limits clinicians’ ability to elect treatment regimens that not only prolong survival but also promote symptom management and high quality of life.

**Methods:** To decipher the heterogeneous presentation of neurologic symptoms in GBM, we investigated differences in the molecular makeup of tumors from patients with and without preoperative neurologic deficits. We used the Ivy GAP (Ivy Glioblastoma Atlas Project) database to integrate RNA sequencing data from histologically defined GBM tumor compartments and neurologic examination records for 41 patients. We investigated the association of neurologic deficits with various tumor and patient attributes. We then performed differential gene expression and co-expression network analysis to identify a transcriptional signature specific to neurologic deficits in GBM. Using functional enrichment analysis, we finally provided a comprehensive and detailed characterization of involved pathways and gene interactions.

**Results:** An exploratory investigation of the association of tumor and patient variables with the early development of neurologic deficits in GBM revealed a lack of robust and consistent clinicopathologic prognostic factors. We detected significant differences in the expression of 728 genes (FDR-adjusted *p*-value ≤ 0.05 and relative fold-change ≥ 1.5), unique to the cellular tumor (CT) anatomical compartment, between neurologic deficit groups. Upregulated differentially expressed genes in CT were enriched for mesenchymal subtype-predictive genes. Applying a systems approach, we then identified co-expressed gene sets that correlated with neurological deficit manifestation (FDR-adjusted *p*-value < 0.1). Collectively, these findings uncovered significantly enriched immune activation, oxidative stress response, and cytokine-mediated proinflammatory processes.

**Conclusion:** Our study posits that inflammatory processes, as well as a mesenchymal tumor subtype, are implicated in the pathophysiology of preoperative neurologic deficits in GBM.

## Introduction

Glioblastoma (GBM), used to refer to Grade-IV astrocytoma, is the most common, aggressive, and malignant form of primary brain tumors in adults. GBM patients typically present a spectrum of generalized or focal neurologic symptoms including headaches, partial or generalized seizures, unilateral or bilateral paresis, hemiplegia, ataxia, visual defects, cognitive impairment, and personality changes (Iacob and Dinca, 2009; Burks et al., 2016; Georgakis et al., 2018). These symptoms persist throughout the course of the disease and worsen following surgical resection, severely limiting day-to-day functions, impacting patients’ quality of life, and even influencing survival outcomes (Martinez et al., 2008; Iacob and Dinca, 2009; Georgakis et al., 2018; Lee et al., 2018).

While preoperative neurologic symptoms are common amongst GBM patients, their form of manifestation is highly variable (Lee et al., 2018). Such variability is believed to arise from the direct and indirect interplay of tumor and patient factors (Kushner and Amidei, 2015). Tumor factors–such as location, size, molecular composition, microvascular proliferation, vascular permeability, and vasogenic edema–can impact local brain architecture and disrupt neuronal connections and functions (Schoenegger et al., 2009; Faivre et al., 2015; Madhusoodanan et al., 2017). Solid GBM tumors contribute to the physical compression and distortion of the central nervous system, while surrounding migrating tumor cells concurrently exacerbate intracranial pressure upon infiltrating neighboring tissue and increasing local swelling (Giglio and Gilbert, 2010). Patient factors–such as gender, lifestyle, pre-existing medical conditions, and genetic predisposition–can also increase the likelihood of developing comorbid neurologic symptoms (Walid, 2008). Whether or not the neurologic effect of such patient factors is separate from or is in concert with cancer remains unclear. Altogether, this suggests the existence of molecular processes in GBM tumors that contribute to neurological signs.

In this study, we examined the heterogeneous manifestation of neurologic symptoms in GBM patients, integrating transcriptome profiling of GBM tumors with clinical data to elucidate the underlying molecular machinery. We specifically focused the analysis on focal neurologic deficits due to their neuroanatomically localizing nature. Our findings serve as a baseline for further validation in follow-up studies, with the hope of ultimately contributing to the design of targeted therapies for GBM patients at higher risk for neurological complications.

## Materials and Methods

### Clinical Data Collection and Processing

Clinical information for GBM patients (*n* = 41) and corresponding tumors (*n* = 42) was downloaded from the Ivy Glioblastoma Atlas Project (GAP) Clinical and Genomic Database^1^ and its partner database^2^ (Puchalski et al., 2018). The Patient Information tab was used to gather the state of neurologic deficit (“yes” or “no”). The neurologic deficit measure, which reflects the manifestation of preoperative and neuroanatomically localizing focal neurologic deficits, was collected during patient intake or initial diagnosis prior to surgery. A summary of select patient and tumor traits, in which we focused on traits previously associated with GBM patient outcomes (Martinez et al., 2008), can be found in Table 1 and Supplementary Table 2. Tumor size was measured in ImageJ from macroscopic images of resected tumors with a provided scale bar (Supplementary Figure 1). Fisher’s exact test was used to evaluate the relationship between neurologic deficit state and categorical clinical variables. The Mann–Whitney–Wilcoxon non-parametric test was used to assess differences in the mean ranks of continuous clinical variables values between the two neurologic deficit state groups.

**TABLE 1:** Select patient and tumor characteristics for analyzed samples.

**Patient characteristics**						
			**Age (years)**	**Survival (days)**	**KPS^∗∗^ score at diagnosis**	**Tumor size (cm^2^)**
**Total**		**Count**	**mean (*SD*)**	**mean (*SD*)**	**mean (*SD*)**	**mean (*SD*)**

	41	57.8 (12.2)	516 (326)	87.8 (11.07)	16.1 (11.9)	
Gender	Male	21	57.43 (14.24)	437.6 (291.0)	90.5 (9.7)	16.5 (14.3)
	Female	20	58.15 (10.01)	595.3 (349.2)	85 (11.9)	15.7 (9.0)
Neurologic deficit	No	23	56.4 (12.05)	516.5 (378.5)	90.4 (9.8)	17.5 (13.4)
	Yes	18	59.6 (12.5)	516.3 (268)	84.4 (12.0)	14.3 (9.7)

**Tumor characteristics**						

Molecular Subtype^*^	Classical	7	Neural	3		
	Classical/ Mesenchymal	4	Neural/ Proneural	2		
	Classical/ Neural	4	Proneural	8		
	Mesenchymal	5	Unidentified	5		
	Mesenchymal/ Neural	3				
Hemisphere	Left	13				
	Right	27				
	Both	1				
Location	Frontal	9	Temporal	14		
	Parietal	14	Occipital	2		
Tumor samples	LE (Leading edge)	7	CT (cellular tumor)	34	CTmvp (microvascular proliferation)	10
	IT (Infiltrating tumor)	7	CTpan (pseudopalisading cells around necrosis)	17		

^*^Molecular subtype was assessed in the Ivy GAP study using the 840 transcripts from the RNA-seq data provided by Verhaak et al. (2010). ^∗∗^KPS, Karnosky Performance Status.

### RNA-Seq Data Collection and Processing

RNA sequencing (RNA-seq) data was collected from the Ivy GAP database that is made publicly accessible by the Allen Institute (© 2015 Allen Institute for Brain Science. Ivy Glioblastoma Atlas Project^2^). RNA-seq data for anatomic structures and putative cancer stem cells includes samples from the 5 major tumor anatomic structures—cellular tumor (CT), pseudopalisading cells around necrosis (PAN), microvascular proliferation (MVP), leading edge (LE), and infiltrating tumor (IT). For each of the 5 tumor anatomic compartments, detailed information on patient ID; corresponding tumor block ID; tumor sample source; and state of neurologic deficit can be found in Supplementary Tables 1A–E. Gene-level read counts for each tumor sample were used for downstream differential gene expression analysis and weighted gene co-expression network analysis (WGCNA). Estimated read counts were initially generated using RSEM (Li and Dewey, 2011). Complete details of the initial RNA-seq data processing pipeline are presented in the “Overview” documentation of the Ivy GAP database^2^.

### Differential Gene Expression Analysis

For each tumor anatomic compartment, we applied the DESeq2 (Love et al., 2014) pipeline in R to identify differentially expressed genes (DEGs) between the two neurologic deficit groups. With 25,873 genes initially, we first discarded low-expression genes (mean read count < 1). We then evaluated the hierarchical structure of the data (multiple samples per patient, difference tumor sample sources, etc.) and whether it can contribute to unbalanced random effects in the model. For each tumor compartment, we demonstrated the independence between tumor sample source (“Anatomic Structure” or “Cancer Stem Cell”) and state of neurologic deficit (“Yes” or “No). We used Fisher’s exact test (*p*-value = $1.05\times 10^{-1}$ for CT; *p*-value = 1.00 for PAN; and *p*-value = $5.04\times 10^{-1}$ for MVP) to confirm independence. LE and IT analyses consisted of samples from anatomic structures only and were therefore independent of sample source. A summary of the occurrence of tumor sample sources in each neurologic deficit group is provided in Supplementary Table 3. We also evaluated the potential for confounding by correlated samples. Sample clustering using Euclidean distance highlighted correlated samples that stem from multiple sampling from within each patient in a group (Supplementary Figure 2). However, the number of per-patient samples in each group was comparable (2.79 ± 1.12 vs. 3.35 ± 1.60 in CT; 1.83 ± 1.33 vs. 2.36 ± 1.43 in PAN; 1.67 ± 1.15 vs. 2.86 ± 0.90 in MVP; 2.50 ± 0.71 vs. 2.20 ± 0.84 in LE; and 3.00 ± 0.00 vs. 3.00 ± 0.71 in IT) (Supplementary Table 1). As a result, tumor sample source and multisampling did not need to be controlled for in the design formula. We applied shrinkage estimation to perform stable estimation for the dispersion and fold-change for each of the 17,375 remaining genes (Love et al., 2014). We finally extracted significantly differentiated genes within each tumor region that met a Benjamini-Hochberg FDR-adjusted *p*-value cut-off ≤0.05 and a relative fold-change ≥1.5. We used UpSet (Lex et al., 2014) to visualize the comparison of DEGs across tumor regions.

### Functional Enrichment and Upstream Analysis

We used Enrichr (Kuleshov et al., 2016) to perform functional enrichment analysis of DEGs and co-expression gene sets. Enrichr was run at default settings, with significant gene ontology (GO) terms and pathways identified using a combined score (Fisher exact test *p*-value and z-score deviation from expected rank) ≥ 10 and a Benjamini-Hochberg adjusted *p*-value ≤ 0.1. GO terms were obtained from “GO Biological Process 2018” and “GO Molecular Function 2018” whereas pathways were obtained from “KEGG 2016,” “WikiPathways 2016,” “Reactome 2016,” and “BioCarta 2016.” Ingenuity Pathway Analysis (IPA^3^) was used to extract potential upstream regulators of target DEGs, taking into account the directionality and intensity of gene expression changes.

### Enrichment for GBM Molecular Subtype Genes

We gathered lists of GBM molecular subtype-predictive genes from 2 sources: (1) The Cancer Genome Atlas (TCGA) transcriptome signature of GBM tumors classified into 4 groups: mesenchymal (MES; # genes = 216); proneural (PN; # genes = 178); neural (NL; # genes = 129); and classical (CL; # genes = 162) (Verhaak et al., 2010) and (2) TCGA’s subtype gene list filtered for genes uniquely expressed by glioma cells and corresponding to 3 groups: mesenchymal (MES; # genes = 5); proneural (PN; # genes = 50); and classical (CL; # genes = 50) (Wang et al., 2017). We separately evaluated the overlap between our list of DEGs and each of the 2 GBM molecular subtype gene lists. We then determined the significance of this overlap using the hypergeometric test with Benjamini-Hochberg correction for multiple comparisons.

### Co-expression Network Analysis

We used CT read counts from both neurologic deficit groups to construct the co-expression network. A max raw read count > 1 filter was applied to discard genes with low expression levels across most tumor samples. We also discarded non-varying genes, to mitigate noise in gene expression data, by only keeping those in the upper 75 percentile. The dataset was then log-transformed to reduce skewness and used as input in the WGCNA R package (Langfelder and Horvath, 2008). We used median-based biweight midcorrelation, for its robustness to outliers, and applied a soft-thresholding power β=6 to construct the signed hybrid network. We then used a dynamic tree cut method to designate gene modules that meet a minimum size of 30 genes, a minimum merging height of 0.25, and module membership k⁢M⁢E≥0.7. We tested the association of network modules with select tumor and patient characteristics using Spearman’s correlation of their eigengenes (first principal component, which can be viewed as the average gene expression of a module). Asymptotic *p*-values were then adjusted for multiple comparisons and significant correlations were extracted using a Benjamini-Hochberg adjusted *p*-value ≤ 0.1. Co-expression network construction details can be found in Supplementary Table 7. GeneMANIA (Warde-Farley et al., 2010) was later used to visualize DEGs within network modules that correlated with the state of neurologic deficit. Gene-gene connections were generated using molecular function gene ontology-based weighting and were set to represent physical protein interactions, pathway involvement, and predicted functional relationships. Functional terms enriched amongst network genes were identified using an FDR ≤ 0.1.

## Results

### Neurologic Deficit Variation in GBM Was Not Fully Explained by Differences in Prognostic Patient and Tumor Variables

**FIGURE 1:**
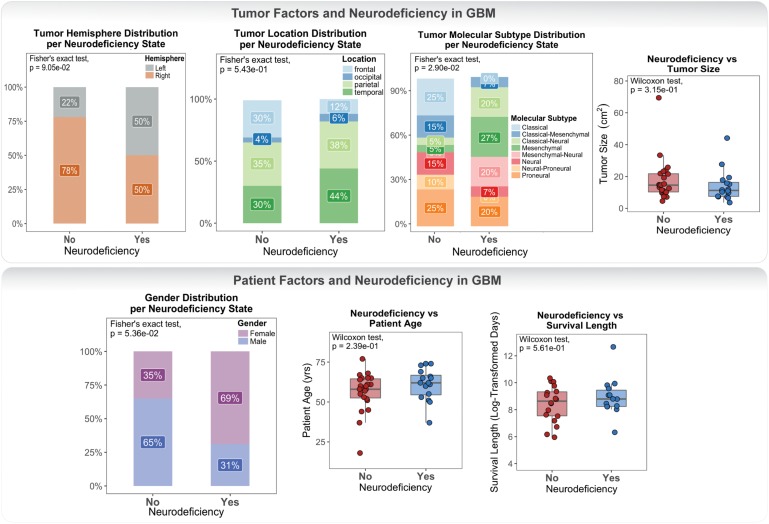
Differences in tumor and patient phenotypic features across glioblastoma (GBM) patients with and without preoperative neurologic deficits.

Clinical data for our GBM cohort was downloaded from Ivy GAP (Material and Methods; Table 1). 41 patients, with a total of 42 tumors, had available neurologic deficit information. We first investigated the association of neurologic deficits with various prognostic clinical attributes that have been previously shown to predict patient outcome in GBM (Figure 1 and Supplementary Table 1). GBM patients with neurologic deficits exhibited a higher rate of left-hemispheric tumors (Fisher’s exact test; *p*-value = $9.05\times 10^{-2}$), with the majority being female (Fisher’s exact test; *p*-value = $5.36\times 10^{-2}$). The distribution of tumor molecular subtypes also varied across patients, with an overrepresentation of mesenchymal tumors in the neurodeficient group and classical tumors in the non-neurodeficient group (Fisher’s exact test; *p*-value = $2.90\times 10^{-2}$). On the other hand, GBM patients with and without neurologic deficits exhibited less differences in tumor location (Fisher’s exact test; *p*-value = $5.43\times 10^{-1}$) and size (Wilcoxon test; *p*-value = $3.15\times 10^{-1}$). They also had a relatively similar distribution of ages (Wilcoxon test; *p*-value = $2.39\times 10^{-1}$) and survival length (Wilcoxon test; *p*-value = $5.61\times 10^{-1}$).

Our association results were limited by statistical power and therefore could not be used to make inferences and draw definite conclusions. They did, however, highlight a lack of well-defined connection between neurologic deficits and patient / tumor factors.

### CT Exhibited the Highest Number of Differentially Expressed Genes and a Strong Inflammatory Signature in the Neurological Deficit Group

We used DESeq2 to perform differential gene expression analysis of tumor sample transcriptomes collected from GBM patients with and without neurological deficits (Material and Methods). The analysis was done separately for samples taken from each of the five tumor anatomic blocks: cellular tumor (CT; 106 samples from 34 tumors); leading edge (LE; 16 samples from 7 tumors); infiltrating tumor (IT; 21 samples from 7 tumors); microvascular proliferation (MVP; 25 samples from 10 tumors); and pseudopalisading cells around necrosis (PAN; 37 samples from 17 tumors). This enabled us to mitigate effects due to inherent intratumor heterogeneity and uncover tumor regions with the strongest association with neurologic deficit.

**FIGURE 2:**
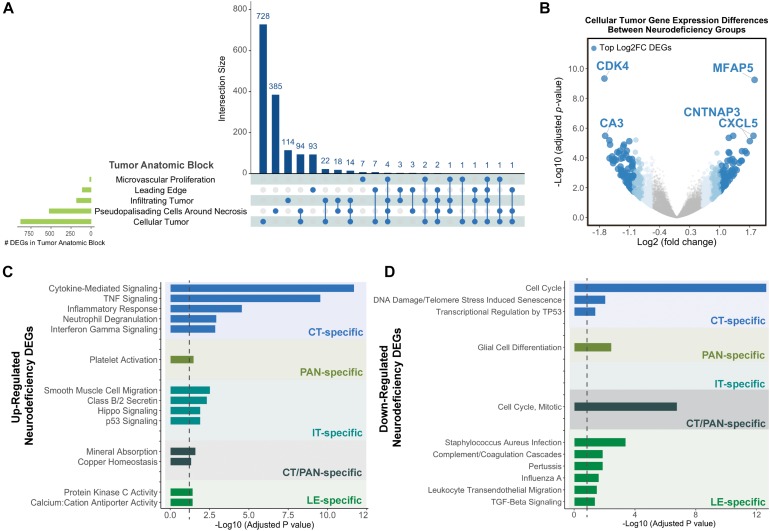
Differential gene expression results for neurodeficiency. **(A)** UpSet diagram showing the intersection size of differentially expressed genes (DEGs; with an FDR-adjusted *p*-value <= 0.05 and |fold change| >= 1.5 cutoff) across tumor anatomic blocks. **(B)** Volcano plot displaying DEGs in the Cellular Tumor (CT) tumor anatomic block and highlighting top fold change (FC) genes. Genes colors are indicated using a quartile-based segmented color scale: not significantly differentially expressed (|fold change| < 1.5); 1.5 ≤ |fold change| < 1.75; 1.75 ≤ |fold change| < 2.0; and |fold change| ≥ 2.0. **(C)** Significantly enriched gene ontology terms and pathways for upregulated tumor anatomic block DEGs, generating using Enrichr. Bar plots indicate statistical significances (adjusted *p*-value). **(D)** Significantly enriched gene ontology terms and pathways for downregulated tumor anatomic block DEGs, generating using Enrichr. Bar plots indicate statistical significances (adjusted *p*-value).

Cellular tumor yielded the highest number of DEGs (*n* = 878), with 728 that did not overlap with other tumor compartments (Figure 2A and Supplementary Tables 3A,C). 154 CT DEGs exhibited over 2-fold relative change in expression levels (Supplementary Table 3B) and included the following top fold-change genes: *CDK4*, *MFAP5*, *CA3*, *CNTNAP3*, and *CXCL5* (Figure 2B). We also detected 385, 114, and 93 tumor compartment-specific DEGs in PAN, IT, and LE, respectively, as well as 94 DEGs that recurred in both CT and PAN (Supplementary Tables 3D–G). We noted the lowest number of compartment-specific DEGs in MVP (*n* = 7) and an overall minimal degree of overlap in DEGs between the distinct tumor anatomic blocks.

**FIGURE 3:**
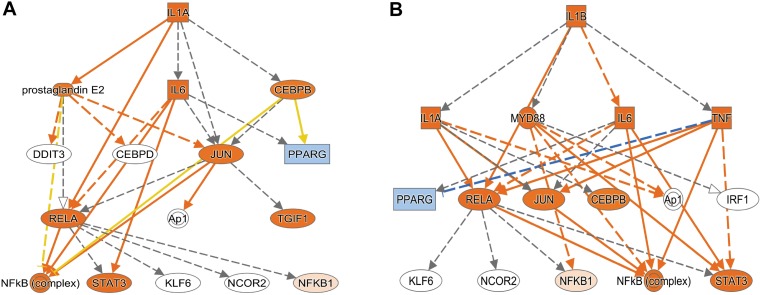
Based on the set of differentially expressed genes in CT, upstream regulator analysis (IPA) predicted an activation of *IL1A* and *IL1B*. **(A)** Results for *IL1A* as potential upstream regulator (*p*-value = 6.54 × 10${}^{-8}$). **(B)** Results for *IL1B* as potential upstream regulator (*p*-value = 2.45 × 10${}^{-6}$). An orange colored node indicates predicted activation, and a blue colored node indicates predicated inhibition. An orange line indicates a regulator leads to activation, and a blue line indicates a regulator leads to inhibition. A yellow line shows findings to be inconsistent with the actual state of the downstream molecule. A gray line indicates an unpredicted effect.

Using Enrichr (Material and Methods), we then functionally categorized gene-level findings from tumor anatomic blocks that harbored the majority of identified DEGs: CT; PAN; IT; CT and PAN; and LE (Figures 2C,D). CT-specific DEGs produced the highest functional enrichment signal, with genes upregulated in the neurologic deficit group revealing a strong inflammatory signature and downregulated genes showing cell cycle-related processes (Supplementary Table 4). To identify potential upstream pathways leading to differential expression in CT, we performed upstream regulator analysis using Ingenuity Pathway Analysis (IPA; Material and Methods). Accounting for the directionality of changes, we identified several potential transcriptional regulators associated with CT-specific differential expression. The full list can be found in Figure 3 and Supplementary Table 5. Top potential regulator genes include interleukin-1 receptor agonists *IL1A* (*p*-value = 6.54 × 10${}^{-8}$) and *IL1B* (*p*-value = 2.45 × 10${}^{-6}$) (Figures 3A,B). An upregulation of those two genes could theoretically elicit a biological cascade leading to the gene expression changes we observed in CT DEGs.

### CT Genes Upregulated in the Neurologic Deficit Group Were Overrepresented in the Mesenchymal Subtype

To further evaluate the relationship between neurologic deficit state and tumor molecular subtype, we scoped out overlaps between our list of CT DEGs and GBM subtype-predictive genes. While Ivy GAP tumor samples were predesignated using TCGA’s list of subtype-predictive genes (Verhaak et al., 2010), 5 out of 41 patients had mismatched subtype assignments between corresponding CT and bulk tumor samples, 13 out of 41 patients had multiple matching subtypes, and 4 out of 41 patients had no subtype assignments (Materials and Methods). The incomplete stratification of GBM patients is attributed to the high degree of intratumor and microenvironment heterogeneity in GBM, with a varying collage of tumor, stroma, normal, and immune cell populations (Bonavia et al., 2011). In addition to TCGA’s list of GBM subtype genes, we therefore also included another list that further filtered for glioma cell-specific genes (Wang et al., 2017) (Materials and Methods).

**FIGURE 4:**
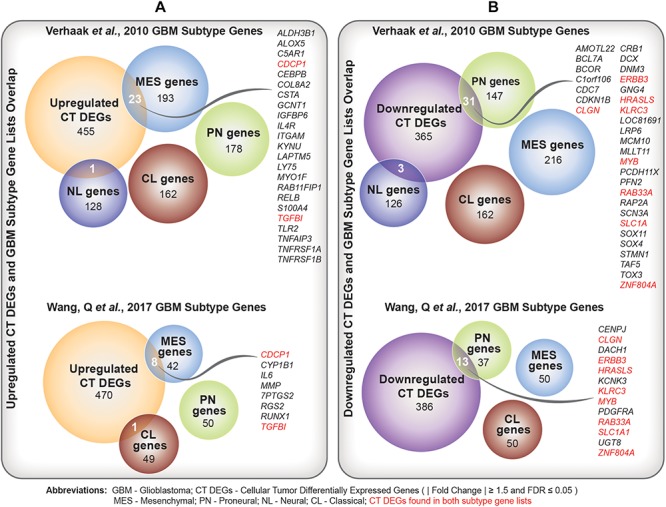
**(A)** Comparison of upregulated Cellular Tumor (CT) differentially expressed genes (DEGs) and two sets of glioblastoma (GBM) molecular subtype signature genes (Verhaak et al., 2010; Wang et al., 2017). Overlapping mesenchymal genes are highlighted. In red are overlapping genes repeated in both sets of GBM subtype genes. **(B)** Comparison of downregulated CT DEGs and two sets of GBM molecular subtype signature genes (Verhaak et al., 2010; Wang et al., 2017). Overlapping proneural genes are highlighted. In red are overlapping genes repeated in both sets of GBM subtype genes.

We identified a significant overlap between upregulated CT DEGs and mesenchymal subtype-predictive genes provided by Verhaak et al. (2010) (*n* = 23; hypergeometric test; FDR-adjusted *p*-value = $4.18\times 10^{-9}$) and Wang et al. (2017) (*n* = 8; hypergeometric test; FDR-adjusted *p*-value = $1.05\times 10^{-5}$) (Figure 4A and Supplementary Table 6A). On the other hand, we noted a significant overlap between downregulated CT DEGs and proneural subtype-predictive genes provided by Verhaak et al. (2010) (*n* = 31; hypergeometric test; FDR-adjusted *p*-value = $2.20\times 10^{-20}$) and Wang et al. (2017) (hypergeometric test; *n* = 13; FDR-adjusted *p*-value = $3.20\times 10^{-12}$) (Figure 4B and Supplementary Table 6B).

### Co-expressed Gene Modules Correlated With Neurologic Deficit Were Implicated in Proinflammatory Cytokines and Oxidative Stress Response

**FIGURE 5:**
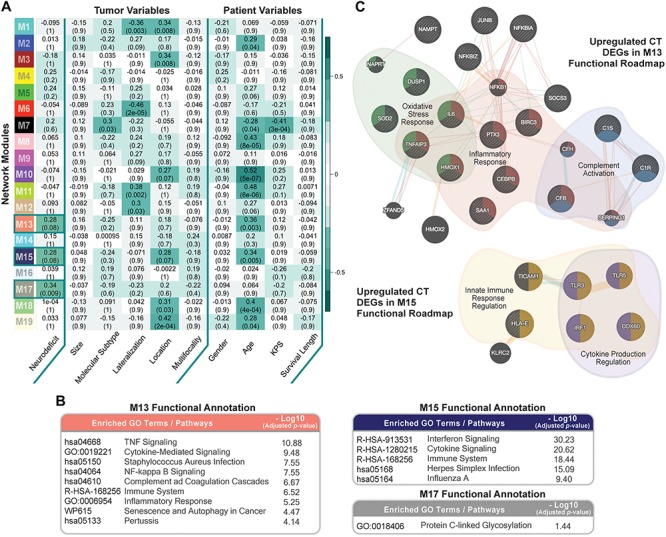
Co-expression network module correlation with neurologic prognostic features. **(A)** Heatmap of the correlation of module eigengenes with select tumor and patient characteristics. Cell values represent the Spearman’s rank correlation coefficient and FDR-adjusted *p*-value for each correlation. Row names (*y*-axis) represent network modules that are color-coded per weighted gene co-expression network analysis (WGCNA) color scheme. Highlighted cells indicate modules that significantly correlate with neurologic deficit state (FDR ≤ 0.1). **(B)** Top Enrichr functional enrichment results for modules M13, M15, M17 that significantly correlated with neurologic deficit. **(C)** GeneMANIA plots depicting the gene-gene functional interactions of upregulated CT DEGs in modules M13 and M15. Edges represent functional connections generated per established information on physical interactions, pathway involvement, and predicted functional relationships. Highlighted are significantly enriched biological processes (FDR ≤ 0.01) identified through GeneMANIA.

We ran weighted gene co-expression network analysis (WGCNA; Material and Methods), using CT samples, to evaluate gene-gene interaction patterns with respect to the variable presentation of neurologic deficits amongst GBM patients. We identified 19 modules and explored their association with prognostic tumor and patient characteristics (Figure 5A). We observed significant positive correlation between modules M13, M15, and M17 and recorded neurologic deficit state (FDR-adjusted *p*-value ≤ 0.1; Figure 5A and Supplementary Table 7).

We then performed functional enrichment analysis using Enrichr (Material and Methods) to gain mechanistic insights into the M13, M15, and M17 neurologic deficit modules. M13 and M15 were significantly enriched for inflammatory processes that include tumor necrosis factor (TNF)-mediated NF-κB activation, cytokine, and interferon signaling (Figure 5B). M17, on the other hand, had a less distinctive functional signature, with only protein C-linked glycosylation as significantly enriched (Figure 5B).

Thirty-one out of 70 genes in M13 overlapped with our previously identified upregulated CT DEGs (Supplementary Table 7). *SOD2* (superoxide dismutase), a CT DEG with a 1.9 times higher expression in the neurologic deficit group, exhibited the highest M13 module interconnectivity (correlation between gene expression level and module eigengene (kME) = 0.911; Supplementary Table 8A). Using GeneMANIA (see section “Materials and Methods”), we demonstrated its significant involvement in oxidative stress response, along with other CT DEGs: *IL6* (interleukin 6); *TNFAIP3* (tumor necrosis factor alpha-induced protein 3); *DUSP1* (dual specificity phosphatase 1); and *HMOX1* (heme oxygenase 1) (Figure 5C). We also observed a central functional role for *NFKBIA* (NF-κB inhibitor alpha; kME = 0.78; Supplementary Table 8B) in inflammatory response and *C1R* (complement C1r; kME = 0.84; Supplementary Table 8B) in complement activation (Figure 5C). In M15, we identified 9 upregulated CT DEGs out of 52 module genes (Supplementary Table 7). A GeneMANIA representation of the functional connections of those genes revealed their involvement in innate immune response and cytokine production regulation (Figure 5C).

## Discussion

Neurologic symptoms in GBM, while varying in clinical manifestation, have a substantial impact on the everyday life of patients. Scientists continue to struggle in deciphering causal mechanisms for the preoperative neurological comorbidity in GBM due to its multifactorial nature, multifaceted clinical presentation, and incomplete documentation. We selected the Ivy GAP GBM cohort, with RNA sequencing information from histologically characterized anatomical tumor compartments and detailed neurological records, to identify factors potentially involved in the pathophysiology of preoperative neurologic deficits in GBM.

We first investigated clinical data to evaluate the relationship between GBM prognostic patient and tumor variables and the manifestation of preoperative neurologic deficits. We did not observe a tumor size-, location-, or survival length-specific association with neurologic deficit. A low preoperative performance and functional status is often perceived to confer poor prognosis in GBM (Chambless et al., 2015; Smedley et al., 2018). Its predictability of survival length, however, continues to be unreliable (Lee et al., 2018). We posit that the missing association between neurologic deficit and survival length, within our cohort and across the wider GBM population, is due to the need to incorporate a combination of multiple clinical attributes to accurately predict patient outcome. This finding can also allude to the lack of specificity in manifested neurological symptoms that can go undetected during the initial visit. This highlights the importance of establishing a standardized, objective, and comprehensive neurologic function assessment tool that can adequately capture the functional state of GBM patients upon diagnosis. On the other hand, we noted a higher prevalence of left-hemispheric tumors, a mesenchymal molecular subtype, and female gender in the neurodeficient group. To the best of our knowledge, a clear connection between lesion lateralization, molecular subtype, patient gender, and cancer-associated neurologic deficits has not been previously reported. As a result of the small size of samples investigated in this analysis, and thus insufficient statistical power, we are unable to further elaborate on these relationships or generalize our findings to the wider GBM population. The three relationships were also weak, with unadjusted *p*-values ranging from $2.90\times 10^{-2}$ to $9.05\times 10^{-2}$. It is therefore necessary to further investigate these findings with a different cohort in order to validate the relationship between clinical traits and neurologic deficits. While unable to draw definite conclusions from this analysis, we are able to illustrate the lack of robust and consistent clinicopathologic prognostic factors for the early development of neurologic deficits in GBM.

We next pursued a transcriptome-based investigation in an attempt to characterize differences in the molecular landscape of GBM tumors corresponding to patients with and without neurologic deficits. We identified the CT tumor anatomic region as harboring the majority of genes significantly differentially expressed between the two neurologic deficits groups, with IL1A (interleukin 1 alpha) and IL1B (interleukin 1 beta) as putative regulators of their expression profile changes. We also noted an overlap between DEGs in CT and PAN and little to none between other tumor anatomic regions. Both CT and PAN comprise neoplastic core tumor cell populations and therefore often cluster together (Puchalski et al., 2018). Functional evaluation of upregulated CT DEGs revealed a strong proinflammatory signal, which was again separately detected when investigating co-expressed gene sets in CT that positively correlated with neurologic deficit. We also demonstrated functional interrelations between upregulated CT DEGs within those co-expressed gene sets and their overlapping involvement in oxidative stress, inflammatory response, and complement activation as well as associated regulatory mechanisms. Collectively, we speculate that tumor cells in GBM may elicit immunological activation and proinflammatory signaling cascades that can contribute to the early development of neurologic deficits. This process is potentially orchestrated by IL1, a well-established mediator of innate immune response and a master regulator of neuroinflammation, through its induction of several proinflammatory cytokines, including TNFA and IL6, and reactive oxygen species (Basu et al., 2004).

The distinct etiology and morphology of GBM tumors has rendered molecular subtyping a major focus in recent medical investigations of GBM (Verhaak et al., 2010; Young et al., 2015). In our exploratory association analysis, we already noted a higher percentage of the mesenchymal subtype in the neurodeficient group. However, given that our results were marginally significant, we wanted to further explore this relationship by comparing our list of CT DEGs with established GBM subtype gene lists. We observed significant enrichment for mesenchymal predictive genes (Verhaak et al., 2010; Wang et al., 2017) within our upregulated CT DEGs. This is in agreement with previous studies, in which mesenchymal tumors have been characterized by an overexpression of inflammatory genes (Zanotto-Filho et al., 2017) that are also overrepresented in our upregulated CT DEGs.

While this study is the first to investigate a molecular signature for the variable presentation of neurologic deficits in GBM, our results are limited by experimental design and thus require confirmation through a follow-up study to ensure reproducibility. The small patient sample size of the cohort (*n* = 41) may lend itself to inherent biases and higher rates of type II error. We were able to mitigate the concern of selection bias by selecting a sample that is highly representative of the entire GBM population. For example, 18/41 (∼43.9%) of our GBM patients had neurologic deficits at first presentation, which matches the 40–60% rate identified in previous epidemiological studies (Lombardi, 2018). We also pursued multiple layers of independent yet complementary analytical approaches to identify a neurologic deficit signature with a higher level of confidence. Furthermore, the small patient pool size in CT (*n* = 34) corresponded to a total of 106 tumor samples. The large tumor sample size lends itself to an easier rejection of a fold-change-based null hypothesis and therefore the identification of genes with less relevance to the biological question at hand. We therefore used both a fold-change and a stringent FDR-adjusted *p*-value cutoff of 0.05 to minimize false positives. A better design for a future study would have to include a set number of samples per donor and a larger number of donors overall. This will improve the study’s statistical power, allowing us to identify, with higher confidence, molecular, patient, and tumor factors implicated in GBM-associated neurologic deficits. Last but not least, the state of neurologic deficits for each patient in the study was gathered from the clinical summary in the Ivy GAP web portal. The summary reflects clinician annotations that are inherently prone to bias and human error. An in-depth investigation of neurologic exam findings from each clinical visit will help ensure that we filtered out unrelated comorbid effects or sporadic input and instead accurately captured preoperative neurologic deficits.

## Conclusion

Our study represents the first multilayered evaluation of the variable presentation of neurologic deficits in GBM. Through a combination of differential gene expression, co-expression, upstream regulation, and functional and gene set enrichment analysis, and were able to identify a proinflammatory repertoire and a mesenchymal subtype in cellular tumor cells of GBM patients with preoperative neurologic deficits.

## Author Contributions

AK performed the clinical associations, co-expression analysis, functional and molecular subtype enrichment, and data interpretation. H-HJ performed the RNA-seq data pre-processing and differential gene expression analysis. NF processed the clinical data and calculated the tumor sizes. KH performed the functional and upstream regulator analysis using IPA. AK, H-HJ, NF, and KH wrote the manuscript. JM supervised the project and provided the input on the manuscript.

## Conflict of Interest Statement

The authors declare that the research was conducted in the absence of any commercial or financial relationships that could be construed as a potential conflict of interest.

## Footnotes

1 http://ivygap.swedish.org/

2 http://glioblastoma.alleninstitute.org/

3 QIAGEN Inc., https://www.qiagenbioinformatics.com/products/ingenuity-pathway-analysis/
