# Supplementary material for: An Inflammatory Landscape for Preoperative Neurologic Deficits in Glioblastoma
Source: Front Genet. 2019 Jun 4;10:488. doi: 10.3389/fgene.2019.00488 (PMC6559211; doi:10.3389/fgene.2019.00488)
Supplement: Supplementary file 9 [file Data_Sheet_1.PDF]

A

B

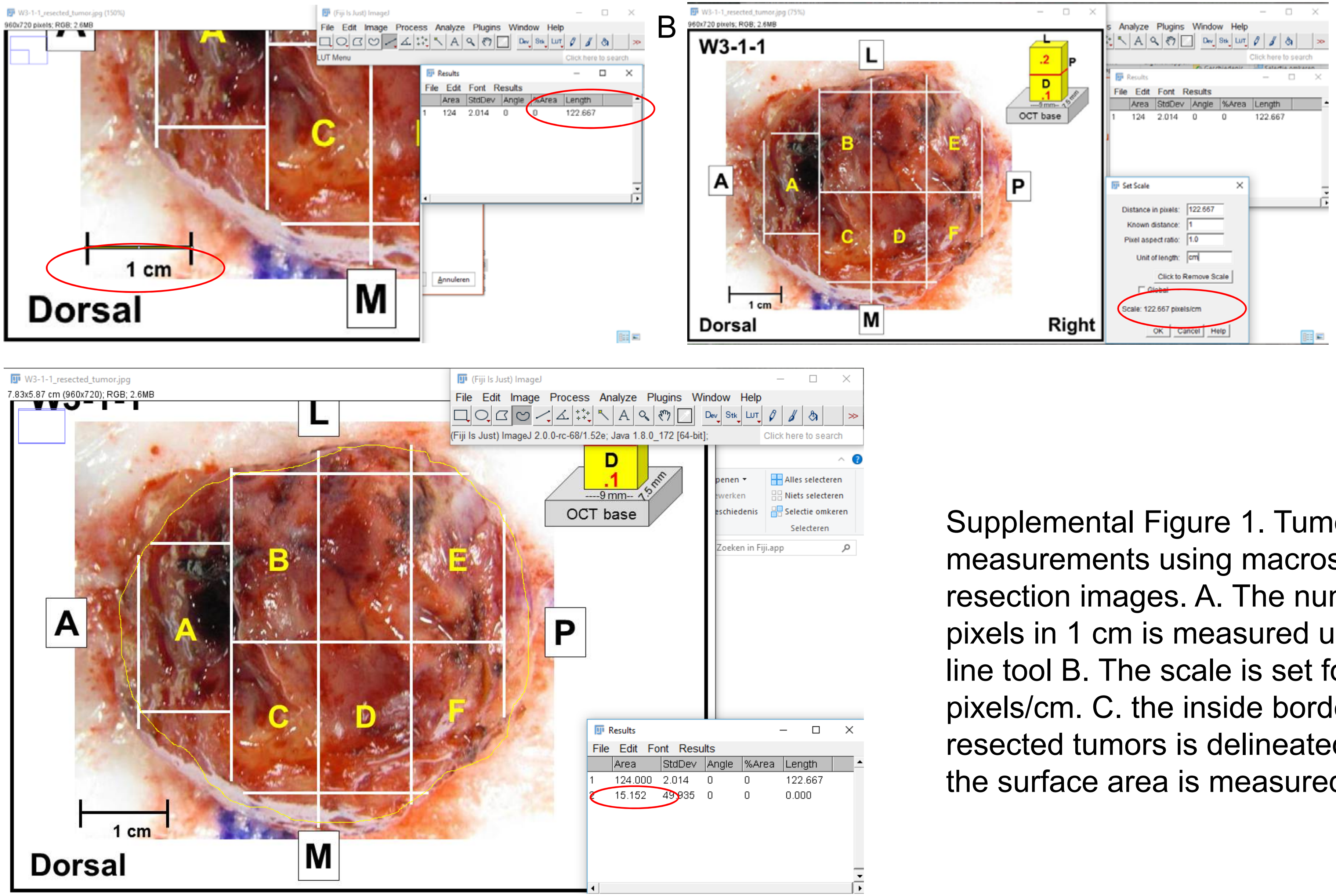

C

Supplemental Figure 1. Tumor size measurements using macroscopic resection images. A. The number of pixels in 1 cm is measured using the line tool B. The scale is set for pixels/cm. C. the inside border of the resected tumors is delineated and the surface area is measured in cm2.
