# Supplementary figures and images for: An Inflammatory Landscape for Preoperative Neurologic Deficits in Glioblastoma
Source: Front Genet. 2019 Jun 4;10:488. doi: 10.3389/fgene.2019.00488 (PMC6559211; doi:10.3389/fgene.2019.00488)

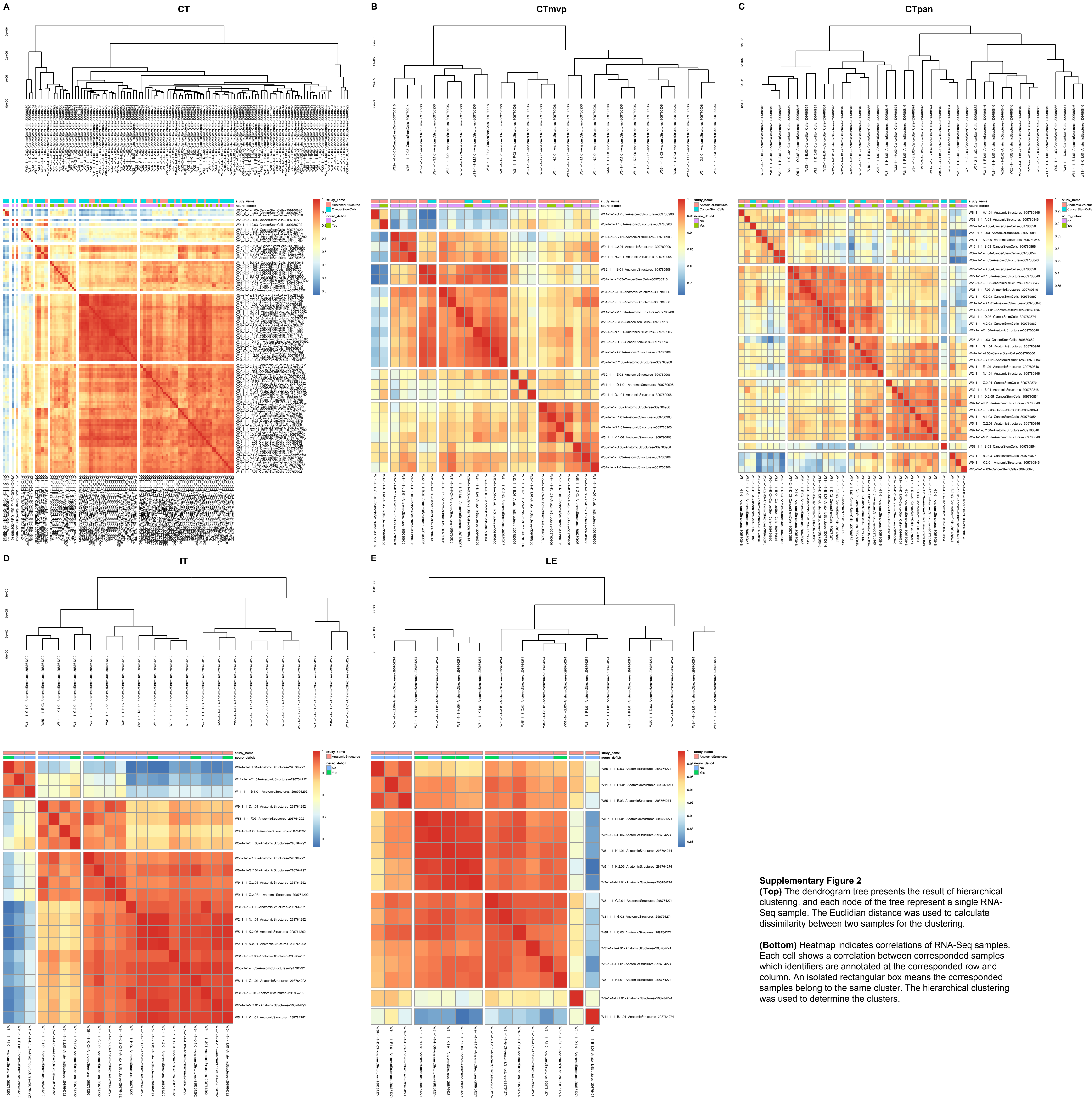

Supplement: Supplementary file 10 [file Data_Sheet_2.PDF]
